# Supplementary material for: STING Orchestrates EV-D68 Replication and Immunometabolism within Viral-Induced Replication Organelles
Source: Viruses. 2024 Sep 29;16(10):1541. doi: 10.3390/v16101541 (PMC11512225; doi:10.3390/v16101541)
Supplement: Supplementary file 1 [file viruses-16-01541-s001.zip › viruses-3119783-supplementary.pdf]

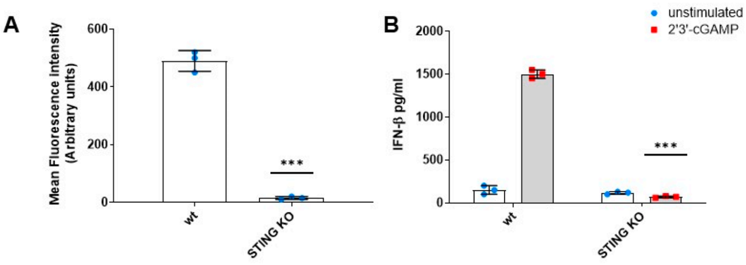

Figure S1. Confirmation of STING deficiency in BEAS-2B STING KO cells. STING knockouts were generated by CRISPR-Cas9 editing in both BEAS-2B. STING deficiency was confirmed using flow cytometry, where no expression of STING protein was detected in STING-knockout (KO) cells (Figure S1A); as well as using a functional assay to stimulate the cells with 2'3'-cGAMP, which is a STING agonist (Figure S1B). Confirmation of STING deficient by flow cytometry (**A**), as well as functional assay (**B**). Data are represented as mean ± SD from three independent experiments. ***, $p < 0.001$.

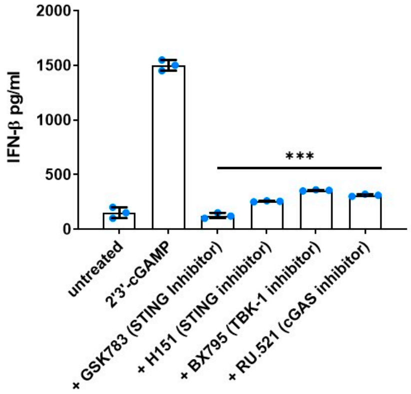

Figure S2. Verification of inhibitor function. Prior to using the inhibitors for pharmacological inhibition of the different molecules, we proceeded to verify that the STING, cGAS and TBK1 antagonists work as intended and inhibit STING-induced IFN-β production (Figure S2). Data are represented as mean ± SD from three independent experiments. ***, $p < 0.001$.

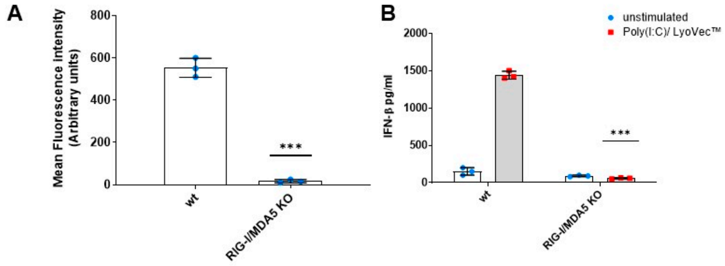

Figure S3. Confirmation of RIG-I/MDA-5 deficiency in BEAS-2B cells. STING knockouts were generated by CRISPR-Cas9 editing in both BEAS-2B. STING deficiency was confirmed using flow cytometry, where no expression of STING protein was detected in STING-knockout (KO) cells (Figure S1A); as well as using a functional assay to stimulate the cells with 2'3'-cGAMP, which is a STING agonist (Figure S1B). Confirmation of RIG-I/MDA5 deficiency by flow cytometry (**A**) as well as functional assay (**B**). Data are represented as mean ± SD from three independent experiments. ***, $p < 0.001$.

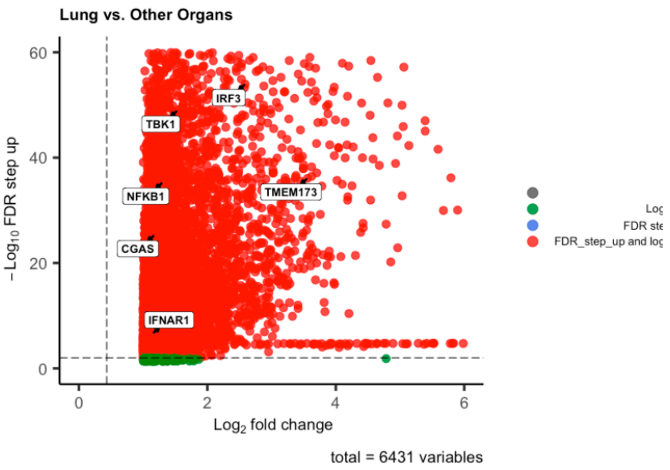

Figure S4. STING (TMEM173) is significantly upregulated following SARS-CoV-2 infection. Single-cell and bulk RNA-seq profiling of COVID-19 patients data revealed significant cGAS-STING signalling pathway genes (TMEM173, IRF3, NFKB1, CGAS, IFNAR1, TBK1) for lung vs all other organs comparison from [24].
